# Microwave-Assisted Polyol Synthesis of Water Dispersible Red-Emitting Eu^3+^-Modified Carbon Dots

**DOI:** 10.3390/ma10010025

**Published:** 2016-12-29

**Authors:** Hailong Dong, Ana Kuzmanoski, Tobias Wehner, Klaus Müller-Buschbaum, Claus Feldmann

**Affiliations:** 1 Karlsruhe Institute of Technology (KIT), Institut für Anorganische Chemie, Engesserstrasse 15, 76131 Karlsruhe, Germany; hailong.dong@kit.edu (H.D.); anan19nana85@hotmail.com (A.K.); 2 Institute of Inorganic Chemistry, University of Würzburg, Am Hubland, D-97074 Würzburg, Germany; tobias.wehner@uni-wuerzburg.de (T.W.); k.mueller-buschbaum@uni-wuerzburg.de (K.M.-B.)

**Keywords:** carbon dot, europium, microwave, polyol, surface conditioning

## Abstract

Eu^3+^-modified carbon dots (C-dots), 3–5 nm in diameter, were prepared, functionalized, and stabilized via a one-pot polyol synthesis. The role of Eu^2+^/Eu^3+^, the influence of O_2_ (oxidation) and H_2_O (hydrolysis), as well as the impact of the heating procedure (conventional resistance heating and microwave (MW) heating) were explored. With the reducing conditions of the polyol at the elevated temperature of synthesis (200–230 °C), first of all, Eu^2+^ was obtained resulting in the blue emission of the C-dots. Subsequent to O_2_-driven oxidation, Eu^3+^-modified, red-emitting C-dots were realized. However, the Eu^3+^ emission is rapidly quenched by water for C-dots prepared via conventional resistance heating. In contrast to the hydroxyl functionalization of conventionally-heated C-dots, MW-heating results in a carboxylate functionalization of the C-dots. Carboxylate-coordinated Eu^3+^, however, turned out as highly stable even in water. Based on this fundamental understanding of synthesis and material, in sum, a one-pot polyol approach is established that results in H_2_O-dispersable C-dots with intense red Eu^3+^-line-type emission.

## 1. Introduction

Carbon dots (C-dots) have recently attracted considerable attention due to their unique properties (e.g., inexpensive nature, chemical stability, adaptable surface functionalization, high biocompatibility, intense photoluminescence (PL)) and a wide range of potential applications (e.g., bioimaging/biosensing, optoelectronics, catalysis) [1,2,3,4,5,6]. Typically, C-dots show intense, broad PL in the blue to green spectral range [7,8,9]. Intense and stable red emission, in contrast, was only reported in few papers [10,11]. Efficient red emission, on the other hand, is most essential for full-colour emission and additive colour mixing to white light, as well as for biomedical application since long-wavelength excitation and emission are less harmful for tissue and show deep-tissue penetration. Finally, green autofluorescence of tissue is avoided [12].

Recently, we could present lanthanide-modified C-dots and their PL for the first time [13,14]. Thus, Eu^3+^- and Tb^3+^-modified C-dots were prepared via the polyol method [15] by in situ thermal decomposition of the solvent (e.g., polyethylene glycol 400/PEG400) and showed excellent quantum yields for line-type red (75%) and green (85%) emission [13]. Meanwhile PEG-modified C-dots are considered as most promising for biomedical applications [16]. Although line-type red *f*-*f* emission of Eu^3+^-modified C-dots is highly promising, several restrictions limit their use by now: (i) the PL is of limited reproducibility; (ii) the emission is rapidly quenched by humidity, which is not acceptable for biomedical application; and (iii) the achievable yield of C-dots is very low.

To protect Eu^3+^ centers against H_2_O-driven quenching, advanced synthesis techniques and sophisticated coordination chemistry were suggested [17,18,19]. Thus, Zhou et al. have modified C-dots by attaching Eu^3+^-coordination complexes with diethylenetriamine pentaacetic acid as a ligand [17]. Song et al. have modified the C-dot surface by self-assembled Eu^3+^ and 5’-guanosine monophosphate [18]. Ye et al. pre-synthesized the coordination complex 4,4′-bis (1″,1″,1″,2″,2″,3″,3″-heptafluoro-4″,6″-hexanedion-6″-yl)chloro-sulfo-*o*-terphenyl-Eu^3+^, which was, thereafter, deposited on the C-dot surface [19]. An alternative approach, moreover, suggested nanocomposites composed of Eu^3+^-doped LaF_3_ and C-dots and results in a quantum yield of 11% [20].

**Figure 1:**
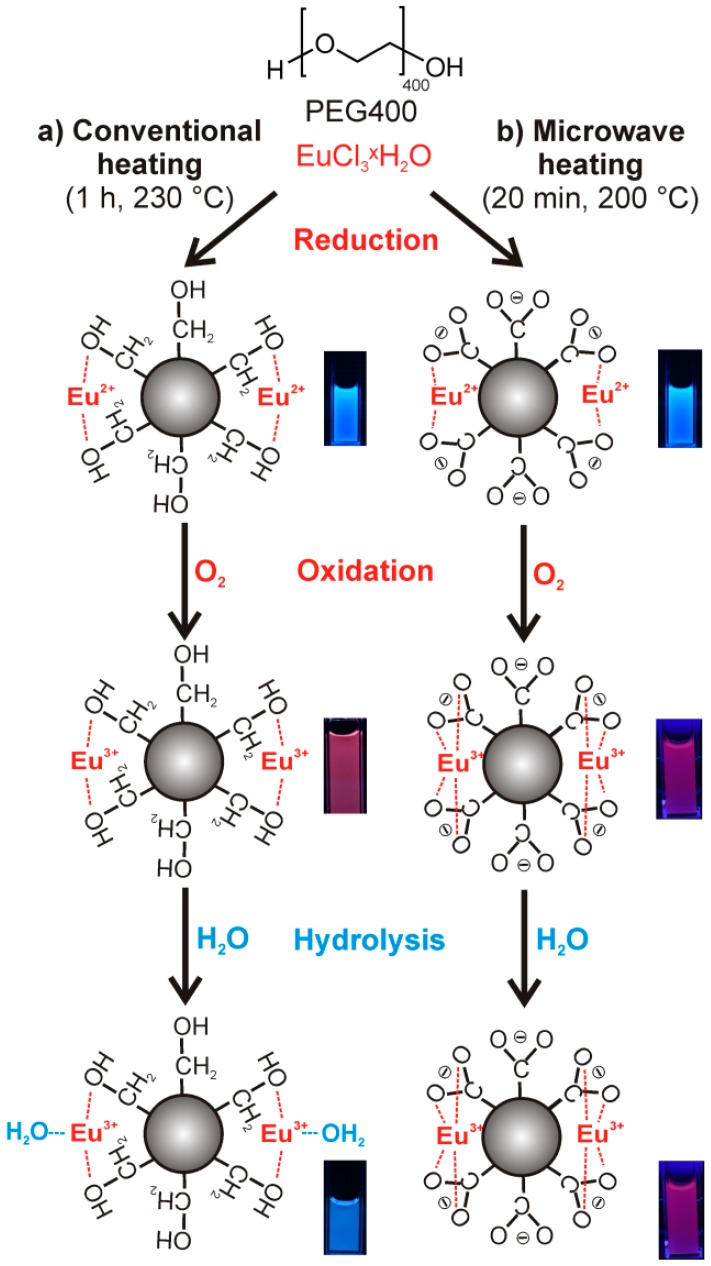
One-pot polyol synthesis of Eu-modified C-dots with the role of Eu^2+^, Eu^3+^, O_2_, H_2_O, and the influence of (**a**) conventional resistance heating and (**b**) MW-heating on the surface functionalization and PL (excitation via UV-LED, *λ_max_* = 365 nm, SI: Figure S2).

Taken together, fundamental understanding of the PL of Eu^3+^-modified C-dots and their stabilization is still lacking but essential for rational material optimization. In the following, we illustrate the role of Eu^2+^/Eu^3+^, the influence of O_2_ and H_2_O as well as the remarkable difference between conventional resistance heating and MW-heating on the surface functionalization and the resulting PL (Figure 1). With this knowhow, a one-pot, MW-mediated polyol synthesis of Eu^3+^-modified C-dots showing stable red emission in water is realized.

## 2. Experimental

### 2.1. Synthesis

*Synthesis of Eu^3+^-modified C-dots via conventional resistance heating:* In a standard recipe for preparing Eu^3+^-modified C-dots, 0.5 mmol of EuCl_3_ × 6H_2_O were dissolved in 10 mL of PEG400. This solution was heated via a heating mantle in a round-bottomed flask to 230 °C in an argon atmosphere [13]. This temperature was maintained for 1 h resulting in a transparent and colloidally stable suspension of Eu-modified C-dots in excess PEG400. Due to the reducing properties of the polyol at high temperature, Eu^3+^ was reduced to Eu^2+^. To study the re-oxidation of Eu^2+^ to Eu^3+^, dry air was bubbled through the as-prepared suspension (30–35 air bubbles per minute). This re-oxidation is slow and proceeds on a timescale of several days. To evaluate the influence of humidity and water, a low amount of water (0.2 mL) was added.

*Synthesis of Eu^3+^-modified C-dots via microwave (MW) heating:* In a standard recipe for preparing Eu^3+^-modified C-dots, 0.2 mmol of EuCl_3_ × 6H_2_O were dissolved in 50 mL of PEG400. This solution was heated via a microwave oven (1200 W, MLS Rotaprep, MLS, Leutkirch, Germany) in a round-bottomed flask to 200 °C in an argon atmosphere. This temperature was reached in 3 min to 200 °C (at 1200 W) and, thereafter, kept for 20 min (at 800 W). MW heating resulted in an opaque suspension of Eu-modified C-dots in excess PEG400. It is to be noted that a shorter MW treatment (e.g., 10 min) results in very limited amounts of C-dots, whereas a longer period of MW treatment (e.g., 30 min) leads to significantly larger C-dots that are known for weak luminescence. In contrast conventional heating, the Eu^3+^-modified C-dots made via MW-heating do not show any sensitivity to humidity and water. Subsequent to synthesis, they can be dispersed in water and—in contrast to the conventional heating procedure—still show Eu^3+^-based red emission. Accordingly, the MW-heated Eu^3+^-modified C-dots can be directly diluted with water resulting in slightly yellowish, colloidally stable suspensions. Alternatively, yellowish powders were collected after centrifugation and washing (i.e., three times redispersion/centrifugation in/from water).

### 2.2. Analytical Tools

Transmission electron microscopy (TEM), high-angle annular dark-field scanning transmission electron microscopy (HAADF-STEM), and energy dispersive X-ray spectroscopy (EDXS) were conducted with a FEI Osiris microscope (FEI, Hillsboro, OR, USA) at 200 kV, equipped with a Bruker Quantax system (XFlash detector). TEM samples of MW-heated Eu^3+^-modified C-dots were prepared by vacuum evaporation of aqueous suspensions at 120 °C on amorphous carbon (lacey-) film-coated copper grids.

X-ray powder diffraction (XRD) was performed with a Stoe STADI-P diffractometer (Stoe, Darmstadt, Germany) operating with Ge-monochromatized Cu-Kα-radiation (*λ* = 1.54178 Å) and Debye-Scherrer geometry.

Fourier-transform infrared spectra (FT-IR) were recorded on a Bruker Vertex 70 FT-IR spectrometer (Bruker, Ettlingen, Germany) using KBr pellets.

Thermogravimetry (TG) was performed with a Netzsch STA 409C instrument (Netzsch, Selb, Germany) applying *α*-Al_2_O_3_ as a crucible material and reference. The MW-heated Eu^3+^-modified C-dots were heated under air to 1000 °C with a rate of 5 K/min. The resulting data were baseline corrected by subtracting the results of a measurement of an empty crucible.

Fluorescence lifetime: The fluorescence lifetimes were obtained as process decay times with an Edinburgh Instruments (FLS920) spectrometer (Edinburgh Instruments, Livingston, UK). The samples were prepared in quartz glass cuvettes under inert-gas atmosphere. The decay times were recorded by time-correlated single-photon counting (TCSPC) with a 375 nm pulsed laser diode or a microsecond flash lamp) with an excitation wavelength of 375 nm. The fluorescence emission was collected at right angles to the excitation source, and the emission wavelength was selected with a monochromator and detected by a single-photon avalanche diode (SPAD). The resulting intensity decays were calculated through tail fit analysis (Edinburgh F900 analysis software). The quality of the fits was evidenced by low *χ^2^* values (*χ^2^* < 1.4).

Fluorescence spectroscopy (FL) and determination of quantum yield: Excitation and emission spectra were recorded using a photoluminescence spectrometer Horiba Jobin Yvon Spex Fluorolog 3 (Horiba Jobin Yvon, Bensheim, Germany), equipped with a 450 W Xenon lamp, double monochromators, Ulbricht sphere, and photomultiplier as the detector (90° angle between excitation source and detector). Determination of the absolute quantum yield was performed as suggested by Friend [21]. First, the diffuse reflection of the sample was determined under excitation. Second, the emission was measured for the respective excitation wavelength. Integration over the reflected and emitted photons in wavelength range of 390–720 nm by use of an Ulbricht sphere allows calculating the absolute quantum yield. Standard corrections were used for the spectral power of the excitation source, the reflection behaviour of the Ulbricht sphere and the sensitivity of the detector. The QY was obtained from dispersion of the Eu^3+^-modified C-dots in H_2_O that were adjusted to an absorbance of 0.1. The sample holder for determining the absolute quantum yield of suspensions in an Ulbricht sphere was constructed according to Friend and is shown in SI: Figure S1 [21].

UV and blue light emitting diodes (UV- and blue-LED): UV-LED and blue-LED light sources were purchased from Zweibrüder Optoelectronics (Zweibrüder Optoelectronics, Solingen, Germany). The UV-LED operates at a wavelength range of 350–380 nm with *λ_max_* = 365 nm (SI: Figure S2a). The blue-LED operates at a wavelength range of 440–500 nm with *λ_max_* = 465 nm (SI: Figure S2b).

## 3. Results and Discussion

### 3.1. Eu-Modified C-Dots via Polyol Synthesis and Conventional Resistance Heating

Following our previous work [13], we started with Eu^3+^-modified C-dots that were prepared by conventional resistance heating of solutions of EuCl_3_ × 6H_2_O in PEG400 (1 h, 230 °C). The resulting C-dots show variable blue and/or red emission depending on the conditions of heating (e.g., duration, temperature, atmosphere, amount of Eu^3+^). Although some samples show excellent quantum yields (75%) in the polyol, they also show rapid PL quenching in water. Moreover, the yield is limited to about 1 mg of C-dots per 20 mL of PEG400. Considering the reducing properties of the polyols at high temperatures (>180 °C) [15] and the comparably-low electrochemical potential of the Eu^3+^→Eu^2+^ reduction (–0.36 V) [22], the variable emission may result from different amounts of Eu^2+^ and Eu^3+^.

**Figure 2:**
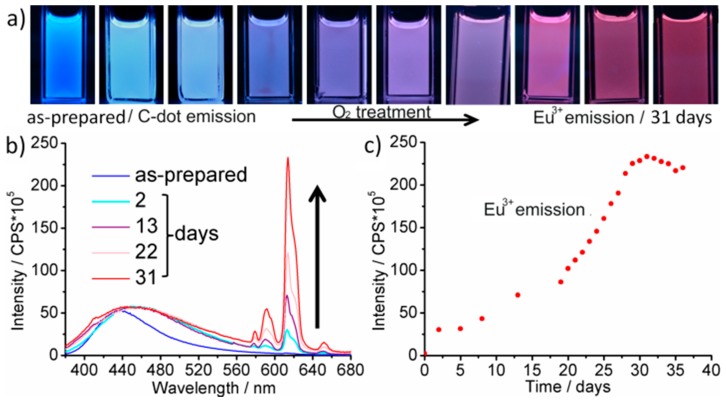
Dry air treatment of conventionally heated Eu-modified C-dots: (**a**) photographs with time-depending emission (UV-LED excitation); (**b**) PL spectra (normalized on C-dot emission at 440 nm); and (**c**) emission intensity of Eu^3+^ at 614 nm (all spectra with *λ_exc_* = 366 nm).

**Figure 3:**
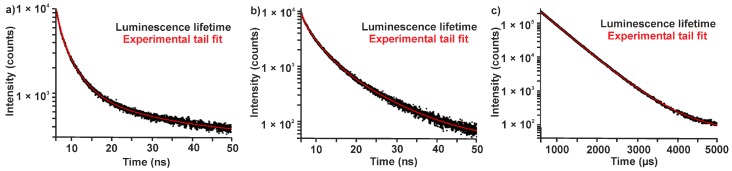
Photoluminescence decay curves Eu-modified C-dots (in PEG400, *λ_exc_* = 375 nm): (**a**) as-prepared (Eu^2+^-modified, *λ_em_* = 440 nm); (**b**) treatment with dry air (2 months, *λ_em_* = 440 nm); and (**c**) treatment with dry air (two months, *λ_em_* = 615 nm).

To elucidate the correlation of synthesis conditions and PL, Eu-modified C-dots were prepared under strict inert conditions (Ar) and, thereafter, stored under Ar (Figure 1a and Figure 2a). Even after two months the suspensions only show the typical broad blue emission of the C-dots upon UV-LED excitation (Figure 2a, SI: Figure S3a). Just a weak peak at 614 nm indicates *f→f* emission of Eu^3+^. Lifetime measurements (*λ_exc_* = 375 nm, *λ_em_* = 440 nm) could be fitted by a multi-exponential equation with decays of *τ*_1_ = 0.6, *τ*_2_ = 2.6 and *τ*_3_ = 11.3 ns (Table 1; Figure 3) that are in good agreement with previously reported lifetime data of C-dots [23,24,25]. It is to be noted that an emission of Eu^2+^ is not to be expected since it would also occur in the blue to green spectral range. Moreover, the C-dot emission is significantly faster (*τ_C-dot_* ~ 1–10 ns) and, therefore, much more efficient than Eu^2+^ emission (*τ_Eu_*_2*+*_ ~ 0.5–0.9 µs) [26].

In contrast to inert conditions, the Eu-modified C-dots in dry air show a slow, continuous increase of the Eu^3+^ *f*-*f* lines and an obvious PL shift from blue to red (Figure 1a and Figure 2a; SI: Figure S3b). In fact, the red Eu^3+^ emission becomes about 25-times more intense than the blue C-dot emission (Figure 2b,c; SI: Figure S4). Whereas the Eu^3+^ emission increases exponentially until saturation with all europium oxidized, the C-dot emission remains at constant level (Figure 2b; SI: Figure S5). Lifetime measurements (*λ_exc_* = 375 nm, *λ_em_* = 440 and 615 nm) again show the typical decay of the C-dots (*τ*_1_ = 0.5, *τ*_2_ = 2.5, *τ*_3_ = 8.4 ns) as well as significantly longer lifetimes (*τ*_1_ = 474.0, *τ*_2_ = 874.6 μs, Table 1; Figure 3) that are indicative of Eu^3+^ emission (*τ_Eu3+_* ~ 500–1000 μs) [27]. Taken together, the observed PL can be rationalized by reduction to Eu^2+^ during the polyol synthesis and subsequent re-oxidation to Eu^3+^ in the presence of oxygen. Only in the presence of Eu^3+^, however, an efficient energy transfer from the C-dots is possible resulting in an intense line-type *f→f* emission.

**Figure 4:**
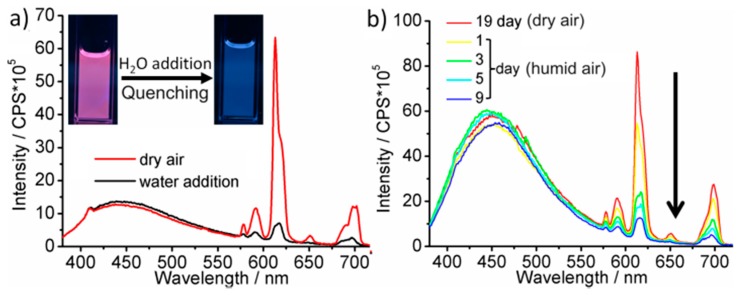
H_2_O-driven quenching of conventionally-heated Eu-modified C-dots: (**a**) photographs and PL spectra with time-depending emission upon addition of water (UV-LED excitation); and (**b**) PL spectra during humid air treatment (*λ_exc_* = 366 nm; normalized on C-dot emission at 440 nm).

It is well-known that the parity-forbidden *f→f* transitions on Eu^3+^ are efficiently quenched by O–H vibrational relaxation, especially, if H_2_O is directly coordinated to the Eu^3+^ center [27]. This effect is here also observed if small portions of water were added to the Eu^3+^-modified C-dots (Figure 4a) or if the as-prepared C-dots were treated with humid air instead of dry air (Figure 4b). In both cases the Eu^3+^ emission at 614 nm is rapidly quenched and only the blue C-dot emission remains (SI: Figure S5). Successive treatment of the Eu-modified C-dots, first, with dry air, and second, with humid air also reproducibly shows an increase (i.e., oxidation to Eu^3+^) followed by a rapid decrease (i.e., H_2_O-driven quenching) of the red emission (Figure 1a; SI: Figure S6).

### 3.2. Eu^3+^-Modified C-Dots via Polyol Synthesis and MW-Heating

**Figure 5:**
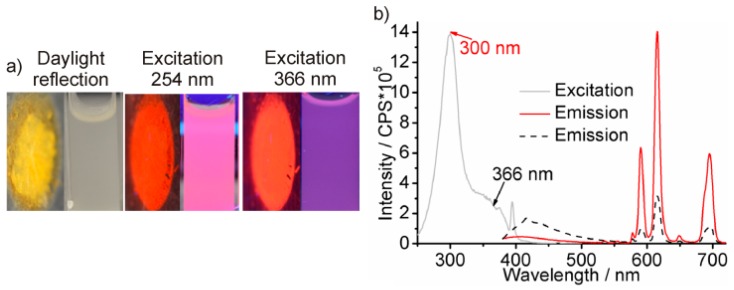
Fluorescence of MW-heated Eu^3+^-modified C-dots: (**a**) photos of powder samples and aqueous suspension (under daylight and under excitation); and (**b**) excitation (*λ_em_* = 615 nm) and emission (red line: *λ_exc_* = 300 nm; black dash: *λ_exc_* = 366 nm) spectra of aqueous suspensions.

After elucidating the role of Eu^2+^/Eu^3+^ and the influence of O_2_/H_2_O on the PL of Eu-modified C-dots, we intended to increase the yield of polyol-made C-dots and to stabilize the Eu^3+^-based red emission, preferentially, in the presence of water. Our previous studies have shown that an increased duration of heating (>2 h) and/or an elevated temperature of heating (>230 °C) indeed support the thermal decomposition of PEG400. Both measures, however, mainly result in a formation of larger C-dots (>10 nm) that only show weak PL [1,2,3,4,5,6,13]. As an alternative to conventional resistance heating we have, therefore, applied MW-heating, which is well known for extremely fast heating, and which is optimal for controlled nucleation of nanoparticles [28]. Hence, a solution of EuCl_3_ × 6H_2_O in PEG400 was MW-heated for 20 min at 200 °C (Figure 1b). With these conditions opaque suspensions were obtained that result in yellowish powders subsequent to centrifugation and washing with 40-times higher yield (40 mg C-dots per 20 mL, Figure 5a). Most importantly, MW-heated, polyol-made Eu^3+^-modified C-dots instantaneously show intense red emission as powder samples (Figure 5a,), and even more interestingly, also after redispersion in water (Figure 5a). This is a remarkable difference of MW-heated and conventionally heated Eu^3+^-modified C-dots.

**Figure 6:**
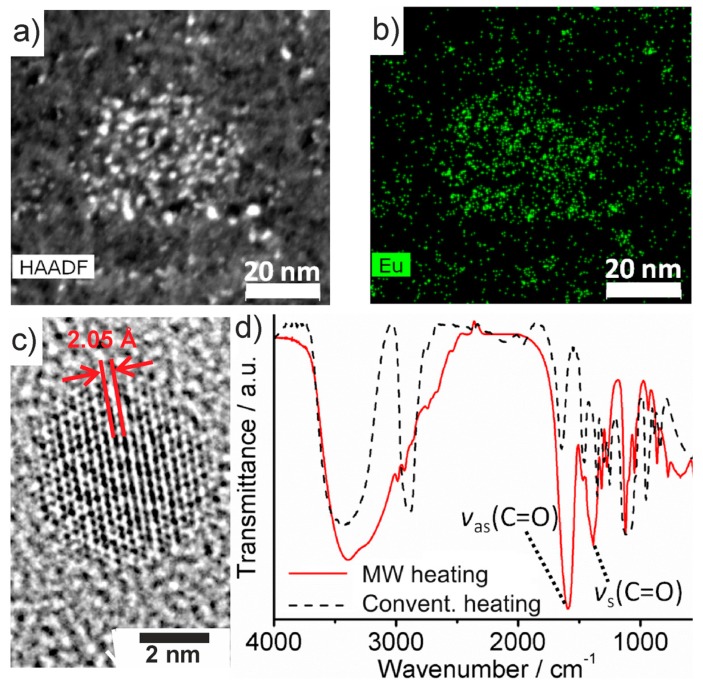
Composition and surface functionalization of MW-heated Eu^3+^-modified C-dots: (**a**) HAADF-STEM image; (**b**) Eu elemental mapping; (**c**) HRTEM image with lattice distance; and (**d**) FT-IR spectra of MW-heated C-dots (**red**) in comparison to conventionally heated C-dots (**black**).

According to high-angle annular dark-field scanning transmission electron microscopy (HAADF-STEM), C-dots made via MW-heating exhibit diameters of 2–4 nm at narrow size distribution (Figure 6a). High resolution (HR)TEM images validate the particle size and the crystallinity of the C-dots (Figure 6c). In fact, this diameter is identical to the size of conventionally heated, polyol-made C-dots [13]. Element mappings, furthermore, indicate a uniform Eu distribution (Figure 6b; SI: Figure S7). Total combustion analysis (thermogravimetry, 1000 °C, air) proves a total carbon content of 68 wt % (SI: Figure S8a). The solid remnant (32 wt %)—according to X-ray diffraction—was identified as Eu_2_O_3_ (SI: Figure S8b). As a result, the Eu^3+^-modified C-dots can be concluded to contain 12 mol % Eu^3+^.

Whereas the particle size of the as-prepared C-dots, the total amount of Eu^3+^ and the C-dot→Eu^3+^ energy transfer with Eu^3+^-based emission were already confirmed, the difference between conventionally heated and MW-heated C-dots and the stable PL of the latter in water remain surprising. To this respect, it is well known that coordination complexes of Eu^3+^ can show intense PL in water if all coordination sites are blocked by stronger ligands preventing energy transfer into vibronic states of water, for instance, by chelating Eu^3+^ with carboxylate ligands [17,18,29,30]. Fourier-transform infrared spectroscopy (FT-IR) indeed indicates a significant difference between the surface conditioning of the polyol-made C-dots (Figure 1 and Figure 6d). Thus, *ν*(O–H) (3600–3000 cm^−1^) represents the dominating vibration for conventional resistance heating, whereas *ν*(C=O) (1700–1400 cm^−1^) is dominating for MW-heated C-dots (Figure 6d). Obviously, the C-dot surface is either hydroxyl-terminated due to polyol functionalization or carboxylate-terminated by oxidized polyols (Figure 6d). Carboxylate-termination of the C-dot surface guarantees for efficient coordination of Eu^3+^ near to the C-dot surface with both fast Eu^2+^→Eu^3+^ oxidation after synthesis and shielding of Eu^3+^ against H_2_O.

Excitation and emission spectra confirm the red emission of MW-heated Eu^3+^-modified C-dots in aqueous suspensions (Figure 5b). For excitation at 366 nm, certain C-dot emission occurs (400–500 nm). Excitation at maximum absorption of the C-dots (300 nm), moreover, shows intense line-type *f*→*f* emission of Eu^3+^ only, indicating efficient Förster resonance energy transfer (FRET) from the C-dot to Eu^3+^. The absolute quantum yield (determined according to *Friend*, SI: Figure 1 and Figure 2) [21] is 18% for aqueous suspensions (*λ_exc_* = 366 nm). Notably, the intense red emission of Eu^3+^-modified, polyol-made C-dots is observed in aqueous suspensions without the need of any additional stabilizing agent.

## 4. Conclusions

A one-pot polyol synthesis of Eu^3+^-modified C-dots is presented. Microwave (MW) heating turned out as a key-factor to increase the yield and to obtain carboxylate-functionalized C-dots that can be directly dispersed in water without complete quenching of the red emission of Eu^3+^. Moreover, the role of Eu^2+^/Eu^3+^, the influence of O_2_/H_2_O and the importance of the surface functionalization on the photoluminescence are correlated and result in a fundamental understanding of synthesis and material properties. Based on this study a knowledge-based synthesis is possible.

Due to their intense line-type red emission, polyol-made Eu^3+^-modified C-dots are highly interesting for optoelectronics (full-colour emission and colour mixing to white light), as well as for optical imaging (sufficient tissue penetration and low background emission for line-type red emission). Finally, the MW-mediated polyol synthesis can be transferred to C-dots showing other rare-earth metal modification and emission.

## Figures and Tables

**Table 1:** Photoluminescence lifetimes of Eu-modified C-dots (suspensions in PEG400, *λ_exc_* = 375 nm).

Sample	*λ_em_*/nm	*B*_1_/%	*τ*_1_/*ns*	*B*_2_/%	*τ*_2_*/ns*	*B*_3_/%	*τ*_3_*/ns*	*χ*^2^
As-prepared (Eu^2+^-modified)	440	14.9	0.57 ± 0.01	45.3	2.56 (±0.02)	39.7	11.31 ± 0.10	1.23
Dry-air treatment (Eu^3+^-modified)	440	11.9	0.53 ± 0.01	37.0	2.52 (±0.03)	51.1	8.43 ± 0.04	1.23
Dry-air treatment (Eu^3+^-modified)	615	93.7	474 × 10^3^ ±1.0	6.3	875 × 10^3^ (±15)	/	/	1.36

*λ_em_*: Emission wavelength at which the decay was monitored; *B*: Percentage contribution of different decay processes; *χ^2^*: Wellness of the exponential fit in comparison to raw data. Values in parentheses indicate the standard deviations of the respective decay time.

## Supplementary Materials

The following are available online at www.mdpi.com/1996-1944/10/1/25/s1. Figure S1: Sample holder for determining the absolute quantum yield of suspensions in an Ulbricht sphere according to Friend. Figure S2: Emission spectra of: (a) UV-LED and (b) blue-LED. Figure S3: Conventionally heated Eu-modified C-dots after storing for 2 month in argon (a), and treatment with dry air thereafter (b) (emission spectra and photographs with *λ*_exc_ = 366 nm). Figure S4: Excitation spectra of Eu-modified C-dots (suspensions in PEG400) made via conventional heating: (a) Storing in argon for 2 months (recorded at *λ*_em_ = 439 nm); (b) Storing in argon for 2 months (recorded at *λ*_em_ = 614 nm); (c) Treatment with dry air for 2 months (recorded at *λ*_em_ = 439 nm); (c) Treatment with dry air for 2 months (recorded at *λ*_em_ = 614 nm). Figure S5: Excitation spectra of Eu-modified C-dots during treatment with dry air: (a) normalized excitation spectra (*λ*_em_ = 445–460 nm); (b) Normalized emission of C-dots at 445–460 nm (*λ*_exc_ = 366 nm). Figure S6: The influence of humidity on Eu-modified C-dots (PEG400 suspensions): (a) Normalized excitation spectra during bubbling of dry and humid air (*λ*_exc_ = 440–460 nm); (b) Emission intensity of Eu^3+^ at 614 nm; (c) Photographs during bubbling of dry (days 0–19) and humid (days 20–28) air (*λ*_exc_ = 366 nm). Figure S7: EDXS images with Eu (a) and C (b) element mappings. Figure S8: Chemical composition of Eu-modified C-dots: (a) Thermogravimetry (TG); (b) XRD pattern of TG-remnant (ICDD 00-034-0392/Eu_2_O_3_ as a reference).
