# Supplementary material for: Microwave-Assisted Polyol Synthesis of Water Dispersible Red-Emitting Eu3+-Modified Carbon Dots
Source: Materials (Basel). 2016 Dec 29;10(1):25. doi: 10.3390/ma10010025 (PMC5344616; doi:10.3390/ma10010025)
Supplement: Supplementary file 1 [file materials-10-00025-s001.pdf]

Supplementary Materials: Microwave-Assisted Polyol Synthesis of Water Dispersible Red-Emitting Eu^{3+}-Modified Carbon Dots

Hailong Dong, Ana Kuzmanoski, Tobias Wehner, Klaus Müller-Buschbaum and Claus Feldmann

1. Analytical Methods

Determination of quantum yield: Determination of the absolute quantum yield was performed as suggested by Friend [1,2]. First, the diffuse reflection of the sample was determined under excitation. Second, the emission was measured for the respective excitation wavelength. Integration over the reflected and emitted photons in a wavelength range of 390–720 nm by use of an Ulbricht sphere allows calculating the absolute quantum yield. Standard corrections were used for the spectral power of the excitation source, the reflection behaviour of the Ulbricht sphere and the sensitivity of the detector. The QY was obtained from dispersion of the Eu^{3+}-modified C-dots in H_2O that were adjusted to an absorbance of 0.1. The sample holder for determining the absolute quantum yield of suspensions in an Ulbricht sphere was constructed according to Friend and is shown in Figure S1 [1,2].

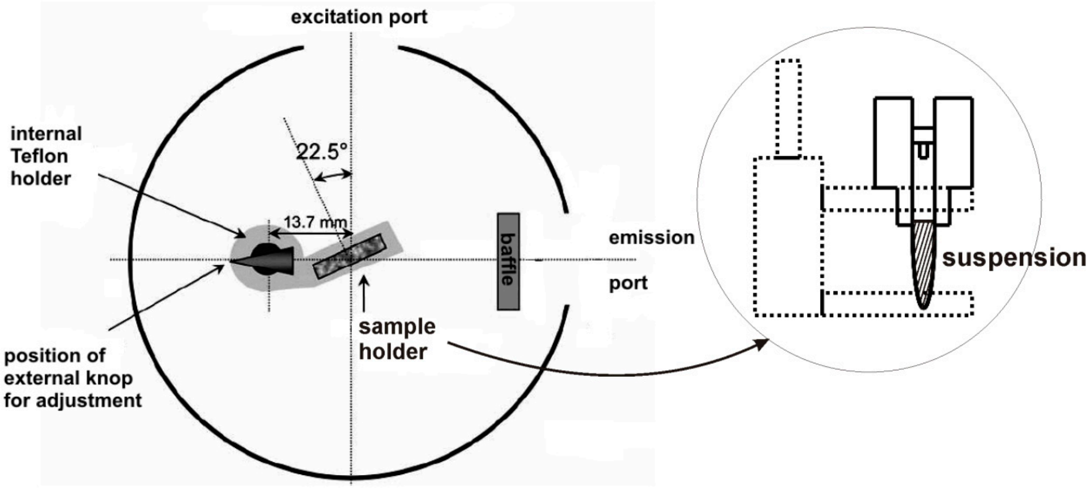

Figure S1. Sample holder for determining the absolute quantum yield of suspensions in an Ulbricht sphere according to Friend [1,2].

UV and blue light emitting diodes (UV- and blue-LED): UV-LED and blue-LED light sources were purchased from Zweibrüder Optoelectronics. The UV-LED operates at a wavelength range of 350–380 nm with λ_{max} = 365 nm (Figure S2a). The blue-LED operates at a wavelength range of 440–500 nm with λ_{max} = 465 nm (Figure S2b).

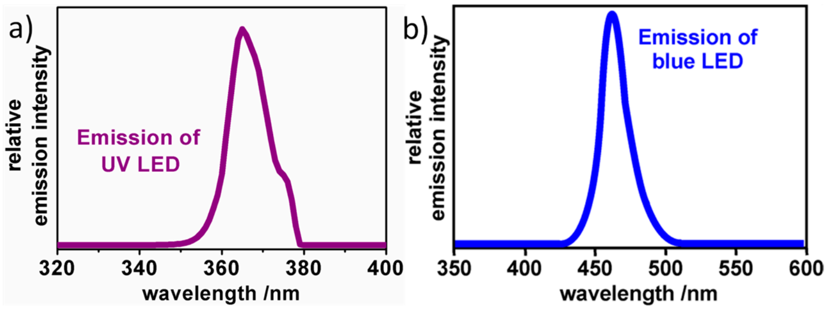

Figure S2. Emission spectra of: (**a**) UV-LED and (**b**) blue-LED.

2. Characterization of Eu-Modified C-Dots Made via Conventional Heating

To elucidate the role of Eu^{3+}/Eu^{2+} and the influence of oxygen, conventionally-heated Eu-modified C-dots were stored under argon for two months (Figure S3a). Thereafter, the suspensions only show the well-known broad C-dot-related emission in the blue spectral region (400–500 nm). The characteristic $f \rightarrow f$ transitions of Eu^{3+} were detected only with weak intensity (614 nm). Photographs of the suspensions under 366 nm excitation show intense blue fluorescence (Figure S3a). This finding again confirms the reduction of Eu^{3+} to Eu^{2+} by the reducing conditions in the heated polyol. If the suspensions were treated with dry air after storing in argon, the intensity of the characteristic $f \rightarrow f$ transitions of Eu^{3+} show a drastically increased intensity with excitation at λ_{exc} = 366 nm (Figure S3b).

The role of Eu^{3+}/Eu^{2+} and the influence of oxygen are also confirmed by excitation spectra of Eu-modified C-dots (in PEG suspension) made via conventional heating (Figure S4). Excitation spectra recorded at maximum emission of the C-dots (λ_{em} = 439 nm) in any case only show the broad excitation of the C-dots between 300 and 400 nm (Figure S4a,c). If excitation spectra were recorded at maximum emission of Eu^{3+} (λ_{em} = 614 nm), the excitation spectra only shows the characteristic $f \rightarrow f$ Eu^{3+} transitions at low intensity after storing under argon (Figure S4b), whereas $f \rightarrow f$ Eu^{3+} transitions are clearly visible with high intensity after treatment with dry air (Figure S4d). The difference between storing under argon and in dry air results in an increase of Eu^{3+}-related emission by a factor of about 25.

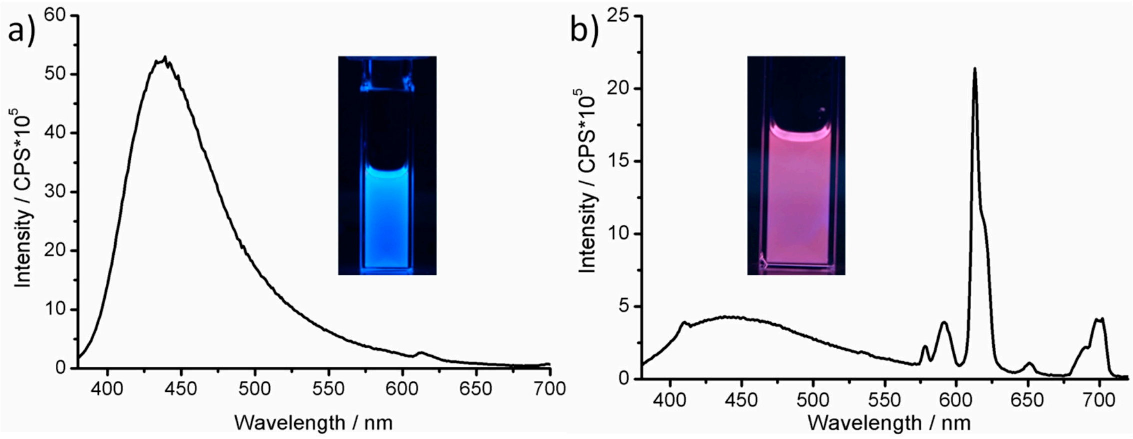

Figure S3. Conventionally-heated Eu-modified C-dots after storing for two month in argon (**a**); and treatment with dry air thereafter (**b**) (emission spectra and photographs with λ_{exc} = 366 nm).

As an additional proof of the reduction of Eu^{3+} to Eu^{2+} during heating in the reducing polyols, lifetime measurements were performed (see main text: Table 1, Figure 3). This comprises two different samples: (a) Eu^{2+}-modified C-dots directly after synthesis with conventional heating; and (b) Eu^{3+}-modified C-dots after treatment with dry air (two months). The lifetime measurements show a clear difference between the Eu^{2+}-modified C-dots—showing decay on a nanosecond timescale (see main text: Figure 3a)—and the Eu^{3+}-modified C-dots—showing a decay on a microsecond timescale (see main text: Figure 3c). Lifetime measurements (λ_{exc} = 375 nm, λ_{em} = 440 nm) were fitted by a multi-exponential equation with decays of τ_1 = 0.6, τ_2 = 2.6, and τ_3 = 11.3 ns for the as-prepared Eu^{2+}-modified C-dots (see main text: Table 1). These values are in good agreement with previously reported lifetime data of C-dots (see main text). A specific decay related to Eu^{2+} is not to be expected since it would also occur in the blue to green spectral range. Moreover, the C-dot emission is significantly faster and, therefore, much more efficient than Eu^{2+} emission (τ_{Eu2+} ~ 0.5–0.9 µs; see main text).

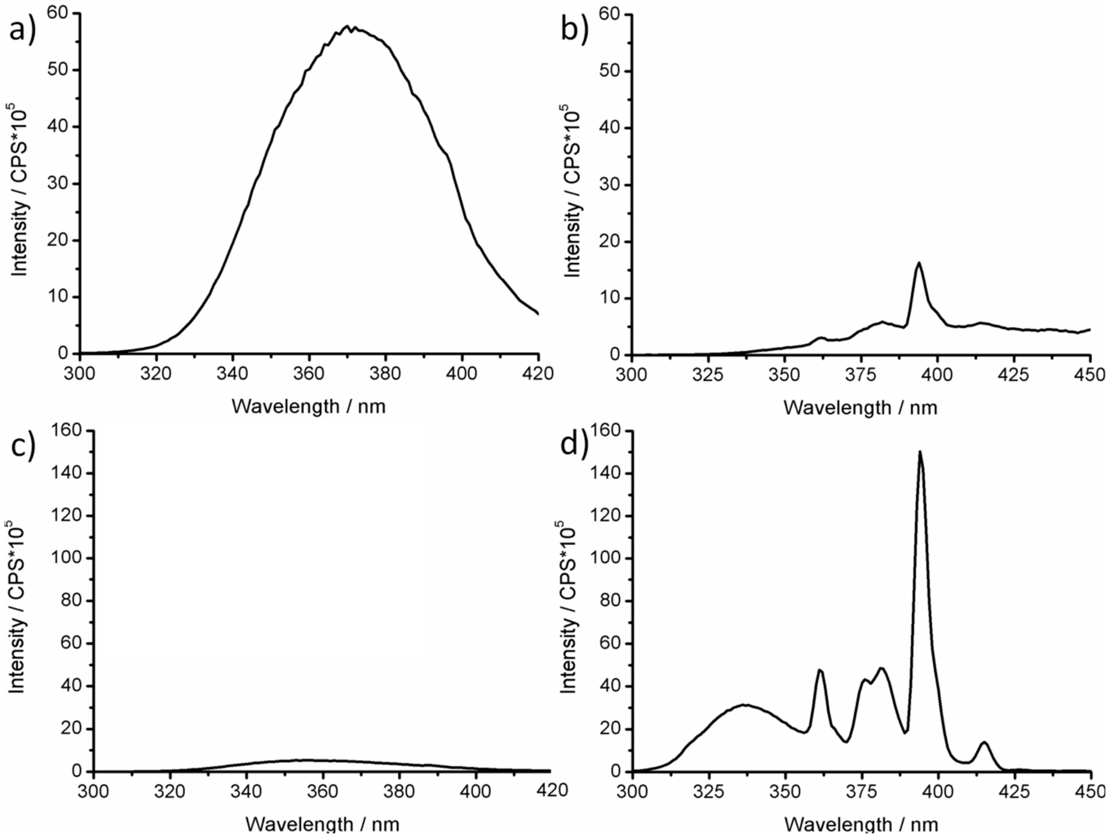

Figure S4. Excitation spectra of Eu-modified C-dots (suspensions in PEG400) made via conventional heating: (**a**) Storing in argon for two months (recorded at λ_{em} = 439 nm); (**b**) storing in argon for two months (recorded at λ_{em} = 614 nm); (**c**) treatment with dry air for two months (recorded at λ_{em} = 439 nm); and (**d**) treatment with dry air for two months (recorded at λ_{em} = 614 nm).

In contrast, the Eu^{3+}-modified C-dots obtained after treatment in dry air clearly show significantly longer lifetimes (λ_{exc} = 375 nm, λ_{em} = 615) of τ_1 = 474.0 and τ_2 = 874.6 µs (see main text: Table 1, Figure 3c) that are indicative for Eu^{3+} emission (τ_{Eu3+} ~ 500–1000 µs, see main text). In addition, the Eu^{3+}-modified C-dots also show short decay (τ_1 = 0.5, τ_2 = 2.5, τ_3 = 11.3 ns) when monitoring the emission of the C-dots (λ_{em} = 440) (see main text: Figure 3b). Thus, the observed photoluminescence can be rationalized by reduction to Eu^{2+} during polyol synthesis and re-oxidation to Eu^{3+} in the presence of oxygen. Only in the presence of Eu^{3+}, however, is an efficient energy transfer from C-dots possible, resulting in intense line-type $f \rightarrow f$ emission (see main text: Figures 1 and 2).

To compare the time-dependent emission of the Eu-modified C-dots while treating with dry air, the respective excitation spectra were normalized on their maximum intensity (Figure S5a). Based on these normalized excitation spectra, the intensity related to the C-dots (blue emission peaking at 445–460 nm) and to Eu^{3+} (peaking at 614 nm) can be illustrated directly. Whereas the C-dot emission remains at constant intensity (Figure S5b), the emission of Eu^{3+} increases significantly over the time (see main text: Figure 2c). This finding can be ascribed to slow oxidation of Eu^{2+} to Eu^{3+}.

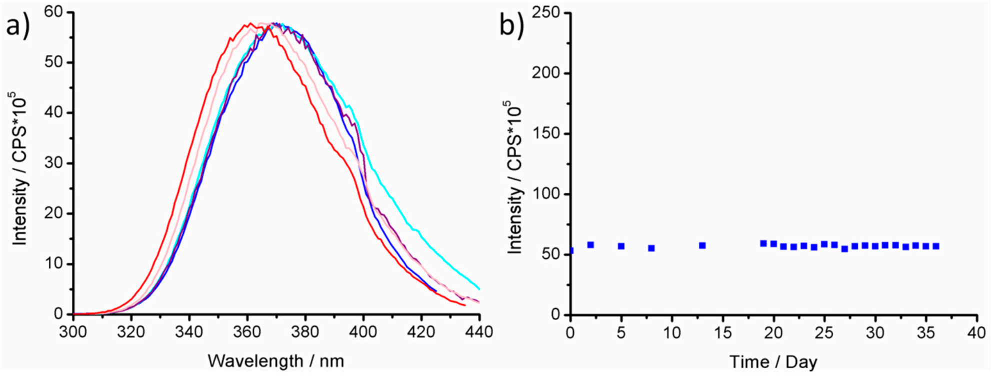

Figure S5. Excitation spectra of Eu-modified C-dots during treatment with dry air: (**a**) normalized excitation spectra (λ_{em} = 445–460 nm); and (**b**) normalized emission of C-dots at 445–460 nm (λ_{exc} = 366 nm).

The influence of humidity is illustrated by treating Eu-modified C-dots with dry air in a first step (for 19 days). As discussed already (Figures S3 and S4; see main text: Figures 1–3), the oxidation Eu^{2+} to Eu^{3+} results in a continuous slow increase of the *f*→*f* transitions of Eu^{3+} (Figure S6). If the dry air is, thereafter,, exchanged by humid air (i.e., air bubbled through water prior to bubbling through the C-dot suspension), the Eu^{3+}-related emission (monitored at 614 nm) drops rapidly due to water-driven fluorescence quenching (Figure S6). For direct comparison, again, the excitation spectra were normalized on maximum intensity (Figure S6a). The emission spectra (Figure S6b) and photographs (λ_{exc} = 366 nm, Figure S6c) show an exponential decrease of the characteristic red Eu^{3+} emission at 614 nm. Due to quenching of the *f*→*f* transitions of Eu^{3+}, finally, only the blue emission of the C-dots remains.

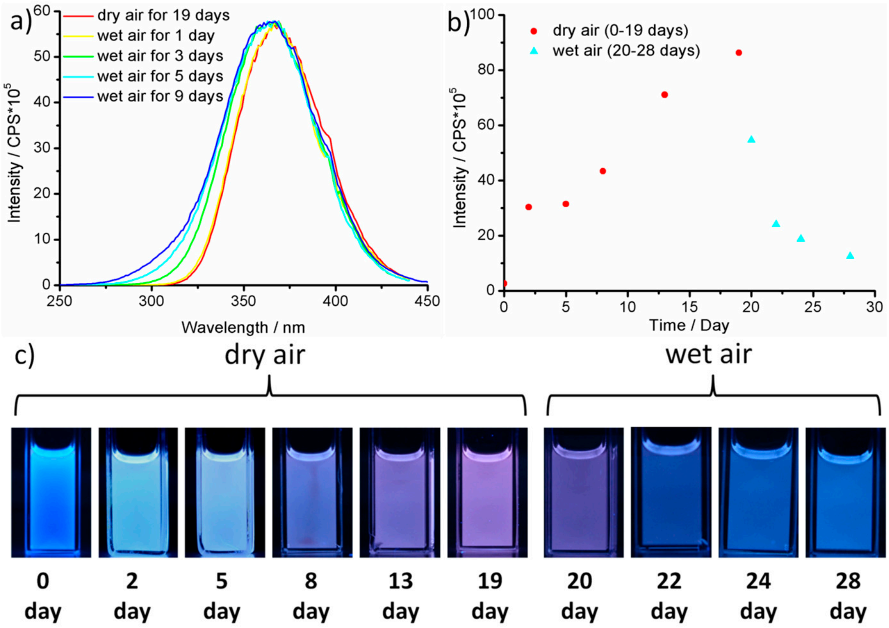

Figure S6. The influence of humidity on Eu-modified C-dots (PEG400 suspensions): (**a**) normalized excitation spectra during bubbling of dry and humid air (λ_{exc} = 440–460 nm); (**b**) emission intensity of Eu^{3+} at 614 nm; and (**c**) photographs during bubbling of dry (days 0–19) and humid (days 20–28) air (λ_{exc} = 366 nm).

3. Characterization of Eu-Modified C-Dots Made via MW Heating

The photoluminescence characterization of MW-heated, Eu-modified C-dots is shown in the main text (see main text: Figure 5). The chemical characterization and electron microscopy of Eu-modified C-dots made via conventional resistance heating is shown in detail in our previous paper [3].

The particle size of Eu-modified C-dots made via conventional heating and MW-heating is identical (3–5 nm, see main text: Figure 6a). Element mappings via energy-dispersive X-ray spectroscopy (EDXS) show a uniform distribution of Eu all over the C-dot surface (Figure S7a; see main text: Figure 6b). Carbon was detected all over the image, which is caused by the TEM grid (i.e., Lacey-carbon coated copper grids, Figure S7b).

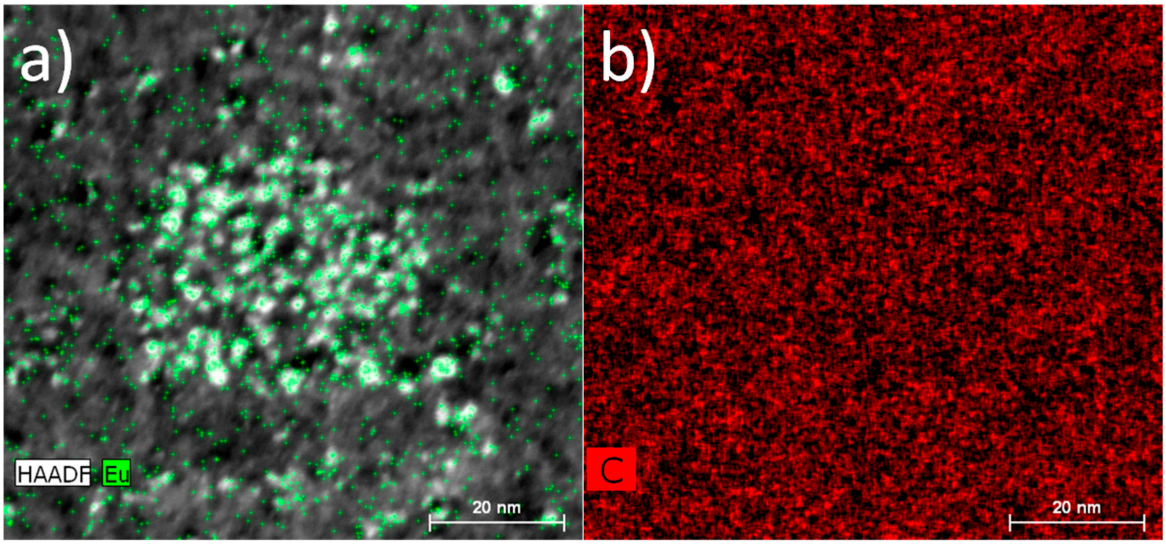

Figure S7. EDXS images with Eu (**a**) and C (**b**) element mappings.

Whereas element mappings clearly indicate the uniform Eu distribution, total combustion analysis (thermogravimetry, 1000 °C, air) indicates a total carbon content of 68 wt % (Figure S8a). The solid remnant (32 wt %), according to X-ray diffraction, was identified as Eu_2O_3 (Figure S8b). Hence, the Eu^{3+}-modified C-dots can be concluded to contain about 12 mol% Eu^{3+} that—according to our previous study [3]—is coordinated on the surface of the C-dots. Taking a particle size of 2–4 nm into account, such a value is to be expected (see main text).

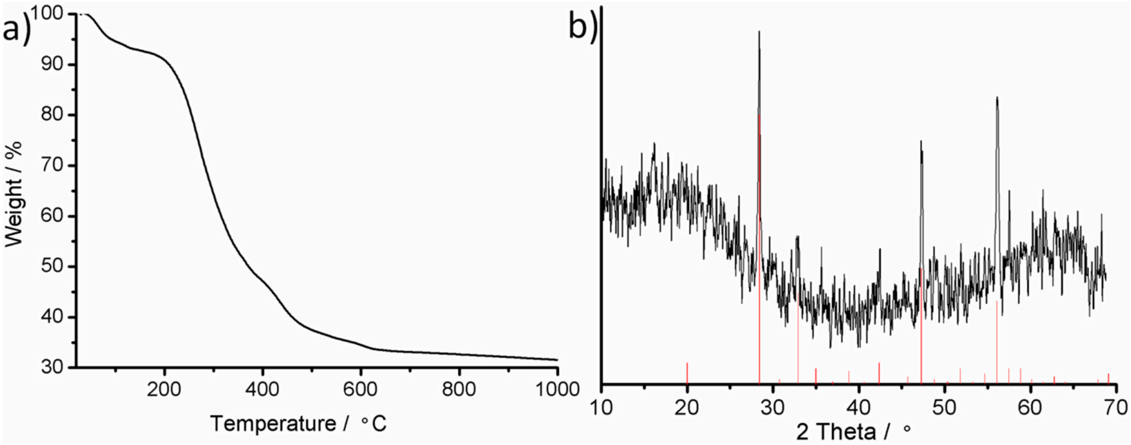

Figure S8. Chemical composition of Eu-modified C-dots: (**a**) thermogravimetry (TG); and (**b**) XRD pattern of TG-remnant (ICDD 00-034-0392/Eu_2O_3 as a reference).

References

1. De Mello, J.C.; Wittmann, H.F.; Friend, R.H. An improved experimental determination of external photoluminescence quantum efficiency. *Adv. Mater.* **1997**, *9*, 230–232.
2. Marks, S.; Heck, J.; Burgos, P.O.; Feldmann, C.; Roesky, P.W. [Ln $(BH_4)_2$ $(THF)_2$](Ln = Eu, Yb)—A highly luminescent material. Synthesis, properties, reactivity, and NMR studies. *J. Am. Chem. Soc.* **2012**, *134*, 16983–16986.
3. Dong, H.; Kuzmanoski, A.; Gößl, D.M.; Popescu, R.; Gerthsen, D.; Feldmann, C. Polyol-mediated C-dot formation showing efficient Tb^{3+}/Eu^{3+} emission. *Chem. Commun.* **2014**, *50*, 7503–7506.
